# Patients and surgeons provide endorsement of core domains for total joint replacement clinical trials

**DOI:** 10.1186/s13075-017-1476-9

**Published:** 2017-12-06

**Authors:** Anh Hoang, Susan M. Goodman, Iris Y. Navarro-Millán, Lisa A. Mandl, Mark P. Figgie, Mathias P. Bostrom, Douglas E. Padgett, Peter K. Sculco, Alexander S. McLawhorn, Jasvinder A. Singh

**Affiliations:** 1 Division of Rheumatology, Hospital for Special Surgery, New York, NY USA; 2 Department of Orthopedic Surgery, Hospital for Special Surgery, New York, NY USA; 3 Medicine Service, VA Medical Center, 510 20th Street South, Faculty Office Tower 805B, Birmingham, AL USA; 4 Department of Medicine, School of Medicine, University of Alabama at Birmingham, 1720 Second Avenue South, Birmingham, AL 35294-0022 USA; 5 Division of Epidemiology, School of Public Health, University of Alabama at Birmingham, 1720 Second Avenue South, Birmingham, AL 35294-0022 USA; 6 University of Alabama at Birmingham, Faculty Office Tower 805B, 510 20th Street South, Birmingham, AL 35294 USA

**Keywords:** Arthroplasty, Outcomes measures, Hip, Knee, Clinical trials

## Abstract

**Background:**

Our objective in this study was to examine whether stakeholders further endorse the core domain set proposed by the Outcome Measures in Rheumatology Trials (OMERACT) total joint replacement (TJR) working group.

**Methods:**

We emailed a survey to 3810 hip/knee arthroplasty patients and 49 arthroplasty surgeons at a high-volume arthroplasty center to rate the importance of each core domain (i.e., pain, function, patient satisfaction, revision surgery, adverse events, and death) and two additional domains (i.e., cost and participation). Ratings were on a 1–9 scale, with 1–3 indicating limited or no importance for patients, 4–6 being important but not critical, and 7–9 being critical. We calculated median (IQR) values and compared ratings by sex, age, and participant type using the Wilcoxon rank-sum test.

**Results:**

The questionnaire was completed by 1295 patients (34%) and 21 surgeons (43%). Patient nonresponders were similar to responders in age (≥55 years, 85.7% vs. 88.6%), sex (female, 57.5% vs. 57.3%), and joint procedure (total hip replacement, 56.9% vs. 63.2%). Overall, all core domains and one noncore domain (i.e., participation) were confirmed as “critical” by both stakeholder groups. Cost was rated as only “important” but not “critical” by surgeons. A completed consensus for all the core domains persisted even when we stratified by sex, age, arthritis type, and the affected joint (knee vs. hip). We received suggestions for additional critical domains from 217 patients and 5 surgeons, prompting the inclusion of 2 research agenda items.

**Conclusions:**

Our study confirmed a consensus rating of the OMERACT TJR core domain set as critical for patients. This broad endorsement should encourage the identification of candidate outcome instruments to further develop a TJR core measurement set that can harmonize reporting in TJR clinical trials.

## Background

Total joint replacement (TJR) is one of the most common and effective elective procedures performed worldwide on patients with end-stage arthritis refractory to medical treatment. The rate of TJR use and its associated health care costs, particularly total hip replacement (THR) and total knee replacement (TKR), are estimated to continue increasing owing to an ever-increasing aging population, the obesity epidemic, and the high prevalence of osteoarthritis (OA) [1–9]. The Outcome Measures in Rheumatology Trials (OMERACT) Total Joint Replacement Working Group previously reported that there is an observed lack of consistency in the outcomes measures and domains used in TJR clinical trials [10–14]. This heterogeneity hampers efforts to perform valid comparisons between TJR clinical trials, including the ability to conduct meta-analyses. Moreover, the need to adhere to the Comprehensive Care for Joint Replacement model makes efforts to harmonize these measures even more prudent [15].

Using a multistep, data-driven process detailed in previous publications [16–20] that mandated the input and consensus of a number of experts and key stakeholders (including patients), as well as the coleadership of orthopedic surgeons, methodologists, and trialists, the OMERACT Total Joint Replacement Working Group proposed six core domains that would help to standardize the reporting of TJR clinical trials. Once the core domain set is widely accepted, a validated measure (or more measures) of each core domain can be identified to create a standardized core measurement set using a data-driven, multistakeholder process similar to the process used earlier for core domains. These six domains, collectively labeled the TJR core domain set, include pain, function, patient satisfaction, revision surgery, adverse events, and death [16, 17]. The core domain set is meant to be reported in every hip/knee TJR clinical trial. The scope of TJR was limited to THR and TKR for this exercise, but it included all end-stage hip and knee arthritis refractory to medical treatment, including OA and rheumatoid arthritis (RA) [17].

The core domain set was recently endorsed by independent groups of orthopedic surgeons and patient partners, providing an important step toward a wider, international multistakeholder consensus [21, 22]. However, a wider dissemination of this domain set to a targeted and relevant audience is still needed. Independent consensus among relevant stakeholders is crucial for the progression of this field so that candidate outcome instruments can begin to be identified for development of a standardized measurement set for TJR. Thus, the objective of this study was to advance the consensus process by querying the same core domain set for two of the most relevant stakeholders: TJR surgeons and TJR patients.

## Methods

We emailed a survey to all eligible patients who had undergone a primary hip or knee TJR in 2015 and had a valid email address available in the electronic health record, as well as to orthopedic surgeons at the Hospital for Special Surgery (HSS), a high-volume orthopedic center of excellence. Patients who had undergone either a bilateral hip or bilateral knee TJR in 2015 or more than one TJR (any joint) in 2015 were excluded. Patients who had undergone subsequent TJRs any time before administration of the survey in 2016 and 2017 were also excluded. To improve the response rate, after the initial administration of the survey, patients and surgeons were sent reminders every week for 3 weeks.

The eligible participants were asked to rate the importance of the six core domains on their own merit, without having to prioritize them. The participants were also asked to rate the importance of two optional areas: cost and patient participation in work and social activities. These additional domains were previous candidates for core domains, but after rounds of deliberations and discussions, these two areas were regarded as noncore [16–18]. Unlike the core domain set, these additional domains were not meant to be reported in every TJR clinical trial. Ratings for each domain were on a 1–9 scale, with 1–3 indicating limited or no importance for patients, 4–6 being important but not critical, and 7–9 being critical. Complete consensus was achieved if both patients and surgeons rated each and every core domain as “critical” (i.e., a rating of 7–9) [21]. Otherwise, it would be considered incomplete consensus and would signal a need to modify the core domain set [21]. In addition to the multiple-choice survey, participants were offered an opportunity to propose additional domains they considered to be critical for TJR clinical trials.

Summary statistics were calculated separately for the TJR patients and TJR surgeons. The median (IQR) ratings were calculated for each of the domains within each group. We also calculated the median (IQR) ratings for the following subgroups of TJR patients: male vs. female, < 55 years vs. ≥ 55 years, OA vs. RA, and THR vs. TKR. We compared ratings between the patients and surgeons, as well as between the different subgroups of patients, using the Wilcoxon rank-sum test. Additional comments proposed by the survey participants were coded into categories using Dedoose software [23]. Ethical approval was provided by the HSS Institutional Review Board (IRB 2017-0040).

## Results

The survey was emailed to 3810 hip/knee arthroplasty patients and 49 hip/knee arthroplasty surgeons. We had to exclude 138 patients who had either a bilateral hip or knee TJR in 2015, 242 patients who had more than one TJR in 2015, and 412 patients who had subsequent TJRs in 2016 and 2017. We received completed questionnaires from 1295 patients (34%) and 21 (43%) surgeons. Patient nonresponders were similar to responders in age (≥55 years, 85.7% vs. 88.6%), sex (female, 57.5% vs. 57.3%), and joint procedure (THR, 56.9% vs. 63.2%). The proportion of male surgeons was > 95% among both responders and nonresponders. The patient cohort had slightly more females (57.3%), whereas the majority of surgeons were male (95.2%) (Table 1). The majority of patients (88.6%) and surgeons (57.1%) were ≥ 55 years of age (Table 1). Within the patient cohort, more respondents had THR procedures (63.2%) and OA (82.7%) (Table 1).

**Table 1:** Characteristics of survey respondents

Category	Patients (*n* = 1295)	Surgeons (*n* = 21)
Female sex	742 (57.3%)	1 (4.8%)
Age ≥ 55 years	1147 (88.6%)	12 (57.1%)
THR	819 (63.2%)	
Osteoarthritis only	1071 (82.7%)	
Rheumatoid arthritis	34 (2.6%)	
Another type of arthritis or joint condition	190 (14.7%)	

*THR* Total hip replacement

Overall, all six core domains were confirmed as “critical” by both patients and surgeons, achieving a median rating of 7, 8, or 9 (Table 2). Patients and surgeons also assessed the two additional domains of cost and participation (not core). Whereas the median score for patient participation was 8 for both groups, cost was rated as only “important” and not “critical” by surgeons (Table 2). The difference in median ratings for cost by the two cohorts was statistically significant (Table 2). A completed consensus for all the core domains persisted even when we analyzed the median ratings among the different patient subgroups (Table 3). All core domains were rated as “critical” when data were stratified by sex, age, arthritis type, and the affected joint (knee vs. hip). Notably, both cost and patient participation were rated as “critical” among all of the patient subgroups (Table 3).

**Table 2:** Domain ratings between patients and surgeons

Core Domains	Overall (*N* = 1316)	Patients (*n* = 1295)	Surgeons (*n* = 21)	*p* Value
Joint pain	9 (8–9)	9 (8–9)	9 (7–9)	0.75
Function or functional ability	9 (8–9)	9 (8–9)	8 (7–9)	0.01
Patient satisfaction	9 (8–9)	9 (8–9)	8 (8–9)	0.02
Revision surgery	8 (5–9)	8 (5–9)	8 (7–8)	0.41
Adverse events	8 (7–9)	8 (7–9)	7 (6–9)	0.23
Death	9 (6–9)	9 (6–9)	9 (7–9)	0.47
Additional domains for consideration	Overall (*N* = 1316)	Patients (*n* = 1295)	Surgeons (*n* = 21)	*p* Value
Cost	7 (5–8)	7 (5–8)	6 (5–6)	0.01
Patient participation in work and social activities	8 (6–9)	8 (6–9)	8 (6–8)	0.26

Each domain was rated on a 1–9 scale, with 1–3 indicating limited or no importance for patients, 4–6 being important but not critical, and 7–9 being critical

**Table 3:** Domain ratings between patient subgroups

	Male	Female	Age < 55 years	Age ≥ 55 years	OA	RA	THR	TKR
Core domains								
Joint pain	8 (7–9)	9 (8–9)	9 (7–9)	9 (8–9)	9 (8–9)	9 (7–9)	9 (8–9)	9 (7–9)
	***						**	
Function	9 (8–9)	9 (8–9)	9 (8–9)	9 (8–9)	9 (8–9)	9 (8–9)	9 (8–9)	9 (8–9)
	***		*		*			
Patient satisfaction	8 (8–9)	9 (8–9)	9 (8–9)	9 (8–9)	9 (8–9)	8 (8–9)	9 (8–9)	8 (8–9)
	***						***	
Revision surgery	7 (4–9)	8 (5–9)	7 (5–9)	8 (5–9)	8 (5–9)	7 (5–9)	8 (5–9)	7 (5–9)
	***							
Adverse events	8 (6–9)	9 (7–9)	8 (7–9)	8 (7–9)	8 (7–9)	8 (5–9)	8 (7–9)	8 (6–9)
	***							
Death	9 (5–9)	9 (6–9)	9 (6–9)	9 (6–9)	9 (6–9)	8 (6–9)	9 (7–9)	9 (5–9)
	***						*	
Additional domains								
Cost	7 (5–8)	7 (5–9)	7 (5–8)	7 (5–8)	7 (5–8)	7 (5–9)	7 (5–8)	7 (5–8)
	***		*					
Patient participation	7 (6–9)	8 (7–9)	8 (6–9)	8 (6–9)	8 (6–9)	8 (7–9)	8 (6–9)	8 (6–9)
	***							

*Abbreviations: OA* Osteoarthritis, *RA* Rheumatoid arthritis, *THR* Total hip replacement, *TKR* Total knee replacement

Significant *p* values are denoted as follows: * *p* ≤ 0.05; ** *p* ≤ 0.01; *** *p* ≤ 0.001. Blank values underneath each pair of median (IQR) indicate no statistical significance. Each domain was rated on a 1–9 scale, with 1–3 indicating limited or no importance for patients, 4–6 being important but not critical, and 7–9 being critical

Of 1295 patient participants, we received suggestions for additional critical domains from 217 (17%). Of these suggestions, 77 (36%) responses were considered replicates of the following existing domains: cost, patient satisfaction, and adverse events. Recovery and rehabilitation time after joint replacement surgery were recommended by 131 (60%) patient respondents (Table 4). In tandem with emphasizing physical recuperation, nine (4%) participants stressed the importance of one’s mental and psychological well-being, both before and after surgery. In contrast, of the surgeon participants, five (24%) suggested additional core domains. Three surgeons highlighted objective physical measurements such as flexibility, gait, and motion. One surgeon echoed the majority of patient responders by emphasizing time to recovery milestones, whereas another surgeon mentioned sport participation and sexual function.

**Table 4:** Characteristics of respondents who provided additional comments

Category	Recovery time (*n* = 131)	Psychological well-being (*n* = 11)	Existing domains (*n* = 77)
Female sex	80 (61.1%)	6 (66.7%)	50 (64.9%)
Age ≥ 55 years	111 (84.7%)	8 (88.9%)	70 (91.0%)
THR	72 (55%)	6 (66.7%)	43 (55.9%)
Osteoarthritis only	118 (90.1%)	4 (44.0%)	61 (79.2%)
Rheumatoid arthritis	3 (2.3%)	4 (44.0%)	1 (1.3%)
Another type of arthritis or joint condition	10 (7.7%)	1 (11.1%)	15 (19.5%)

*THR* Total hip replacement

## Discussion

Researchers in TJR clinical trials have reported heterogeneous outcomes that make it challenging if not impossible when efforts are made to combine and compare outcomes of various implants, surgical techniques, or other interventions across studies in a systematic review, meta-analysis, or comparative analysis [10, 11]. In an era of evidence-based medicine and rising health care costs, meta-analyses are important tools that not only allow comparative analyses but also can help establish the best practices and recommendations for patient care in the peri- and postoperative periods in patients undergoing TJR. A collaborative initiative among international registries has begun to establish a framework to start harmonizing outcome collection and reporting to facilitate value-based health care improvements in the treatment of hip and knee OA [24]. However, such an effort has not yet been completed for hip/knee TJR clinical trials.

Our study helps advance these harmonizing efforts by providing data that demonstrate broad endorsement and consensus of the core domains by both patients undergoing TJR and surgeons performing these procedures. Without endorsement by these key stakeholders, meaningful uptake and adoption of these core domains would be unlikely. Core domains are slated to be reported in every clinical trial, regardless of the intervention and primary outcome. It is important to clarify that core domains and primary outcomes are not synonymous. Depending on the nature of the study, core domain measures may not be the defined primary outcome; the choice of primary study outcome will always depend on the study question. Reporting of core domains in all arthroplasty trials also does not mean that researchers cannot choose other secondary outcomes of relevance to their research question.

**Fig. 1:**
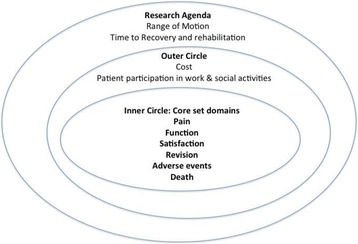
Revised core domain set for total joint replacement clinical trials. The three layers of the “onion” represent the six core domains in the center (*inner circle*), which must be measured in every total joint replacement clinical trial; the middle layer (*outer circle*), consisting of the domains cost and patient participation in work and social activities; and the outermost layer (research agenda), which includes domains of range of motion and the time to recovery and rehabilitation

In this study, patient participation was suggested as a critical domain by both patients and surgeons. This result contrasts with that of past surgeon groups in which patient participation was consistently ranked as only “important” [21]. It may be worth considering patient participation in the future as a core domain; however, it is already included as an additional domain in the current TJR domain set. We recommend that it be included as an outcome in studies focused on improving participation. Meanwhile, cost was critical only to patients, consistent with past observations in smaller cohorts [21, 22]. This difference between patients and surgeons suggests that cost should be included as an additional domain in studies purporting to be patient-centered. Interestingly, the patients’ free text comments strikingly echoed past patient cohorts’ proposals on recovery and rehabilitation, suggesting that further discussions are needed on whether these concepts should be included as important domains for TJR clinical trial reporting in the future [22]. As a result of this study, we believe that it is prudent to add this domain to the research agenda ring of the current OMERACT domain set, which now expands the current “onion” of core outcomes for TJR clinical trials (Fig. 1). The core domain measures proposed by the surgeons (i.e., objective measures of gait and physical function) are already included in the current proposed core domain set that includes function, a domain that can be measured with objective and/or subjective measures. Similarly, flexibility is partially included in the domain of range of motion, which is included in the outermost ring under research agenda. Mental and physical recovery is already captured in the following existing core domains of pain, function, and patient satisfaction.

A notable strength of this study is the administration of the same survey to a large sample size of patient participants who have undergone primary TJR procedures, ensuring appropriate input by those most affected by TJR clinical trials. However, because our sample size included only patients who have undergone knee or hip procedures at a single, large tertiary orthopedic referral hospital, these conclusions cannot be generalized to other types of joint replacements or other hospital settings. Because we required a valid email address for patients among our inclusion criteria, findings may not be generalizable to all patients undergoing TJR. Moreover, because we did not routinely collect data on race, ethnicity, and income or other socioeconomic status markers, we could not stratify and make conclusions about how different subpopulations may have differing opinions and priorities. However, in a previous study, we demonstrated that expectations do not differ between African American and white individuals at HSS [25]. For all of the core domains, the ratings were consistent with that of other patients and surgeon cohorts previously reported in the published literature, corroborating past results [17, 18, 21, 22]. However, considering the number of eligible participants, our response rate was relatively low. Yet, nonresponder and responder characteristics were similar enough to provide confidence in our findings. Owing to a large sample size, even small differences are statistically significant in some cases, even when median scores are the same. These findings should be interpreted with caution.

## Conclusions

Our study confirms that both TJR surgeons and TJR patients agree that the OMERACT TJR core domains are of critical importance as outcome measures in TJR clinical trials. These results support a broad endorsement of, and encourage the identification of, candidate outcome instruments to develop a TJR standardized measurement set.

## Abbreviations

ACR: American College of Rheumatology
HSS: Hospital for Special Surgery
OA: Osteoarthritis
OMERACT: Outcome Measures in Rheumatology Trials
RA: Rheumatoid arthritis
THR: Total hip replacement
TJR: Total joint replacement
TKR: Total knee replacement
