# Correction to: Patients and surgeons provide endorsement of core domains for total joint replacement clinical trials

**DOI:** 10.1186/s13075-018-1509-z

**Published:** 2018-02-14

**Authors:** Anh Hoang, Susan M. Goodman, Iris Y. Navarro-Millán, Lisa A. Mandl, Mark P. Figgie, Mathias P. Bostrom, Douglas E. Padgett, Peter K. Sculco, Alexander S. McLawhorn, Jasvinder A. Singh

**Affiliations:** 1 Division of Rheumatology, Hospital for Special Surgery, New York, NY USA; 2 Department of Orthopedic Surgery, Hospital for Special Surgery, New York, NY USA; 3 Medicine Service, VA Medical Center, 510 20th Street South, Faculty Office Tower 805B, Birmingham, AL USA; 4 Department of Medicine, School of Medicine, University of Alabama at Birmingham, 1720 Second Avenue South, Birmingham, AL 35294-0022 USA; 5 Division of Epidemiology, School of Public Health, University of Alabama at Birmingham, 1720 Second Avenue South, Birmingham, AL 35294-0022 USA; 6 University of Alabama at Birmingham, Faculty Office Tower 805B, 510 20th Street South, Birmingham, AL 35294 USA

## Correction

Unfortunately, after publication of this article [1], it was noticed that Table 2 was not correctly formatted during the production process. The corrected Table 2 can be seen below and the original article has been updated to match.

**Table 2:** Domain ratings between patients and surgeons

	Overall (*N* = 1316)	Patients (*n* = 1295)	Surgeons (*n* = 21)	*p* Value
Core domains				
Joint pain	9 (8–9)	9 (8–9)	9 (7–9)	0.75
**Function or functional ability**	**9 (8–9)**	**9 (8–9)**	**8 (7–9)**	**0.01**
**Patient satisfaction**	**9 (8–9)**	**9 (8–9)**	**8 (8–9)**	**0.02**
Revision surgery	8 (5–9)	8 (5–9)	8 (7–8)	0.41
Adverse events	8 (7–9)	8 (7–9)	7 (6–9)	0.23
Death	9 (6–9)	9 (6–9)	9 (7–9)	0.47
Additional domains for consideration	Overall (*N* = 1316)	Patients (*n* = 1295)	Surgeons (*n* = 21)	*p* Value
**Cost**	**7 (5–8)**	**7 (5–8)**	**6 (5–6)**	**0.01**
Patient participation in work and social activities	8 (6–9)	8 (6–9)	8 (6–8)	0.26

Each domain was rated on a 1–9 scale, with 1–3 indicating limited or no importance for patients, 4–6 being important but not critical, and 7–9 being critical

Bold text signifies results that were statistically significant

## References

[1] Hoang A, Goodman SM, Navarro-Millán IY, Mandl LA, Figgie MP, Bostrom MP, et al. Patients and surgeons provide endorsement of core domains for total joint replacement clinical trials. Arthritis Res Ther. 2017;19:267. 10.1186/s13075-017-1476-9. 10.1186/s13075-017-1476-9 PMC5718077 29208013

